# An Enhanced Adaptive Management Approach for Remediation of Legacy Mercury in the South River

**DOI:** 10.1371/journal.pone.0117140

**Published:** 2015-02-09

**Authors:** Christy M. Foran, Kelsie M. Baker, Nancy R. Grosso, Igor Linkov

**Affiliations:** 1 United States Army Engineer Research and Development Center Duty Station: U.S. Army Corps of Engineers New England District, Concord, MA 01742, United States of America; 2 Contractor for the United States Army Engineer Research and Development Center, Concord MA 01742, United States of America; 3 DuPont Corporate Remediation Group, Wilmington, DE 19805, United States of America; NERC Centre for Ecology & Hydrology, UNITED KINGDOM

## Abstract

Uncertainties about future conditions and the effects of chosen actions, as well as increasing resource scarcity, have been driving forces in the utilization of adaptive management strategies. However, many applications of adaptive management have been criticized for a number of shortcomings, including a limited ability to learn from actions and a lack of consideration of stakeholder objectives. To address these criticisms, we supplement existing adaptive management approaches with a decision-analytical approach that first informs the initial selection of management alternatives and then allows for periodic re-evaluation or phased implementation of management alternatives based on monitoring information and incorporation of stakeholder values. We describe the application of this enhanced adaptive management (EAM) framework to compare remedial alternatives for mercury in the South River, based on an understanding of the loading and behavior of mercury in the South River near Waynesboro, VA. The outcomes show that the ranking of remedial alternatives is influenced by uncertainty in the mercury loading model, by the relative importance placed on different criteria, and by cost estimates. The process itself demonstrates that a decision model can link project performance criteria, decision-maker preferences, environmental models, and short- and long-term monitoring information with management choices to help shape a remediation approach that provides useful information for adaptive, incremental implementation.

## Introduction

Adaptive management has been presented as a way to change or update courses of action based on emerging information to improve the outcome and reduce the uncertainty. It was introduced more than 30 years ago and advanced under the name “adaptive environmental assessment and management” [1–3]. Adaptive management efforts have largely focused on resource management projects, such as the Comprehensive Everglades Restoration Plan (CERP) and the Louisiana Coastal Area (LCA) Program [4–6], while application to contaminated sites has been limited and conceptual in nature [7, 8]. These applications have been the subject of multiple National Academies of Science reports, including one general review [9–11]. One of the most important findings of the review is that adaptive management has regularly been inappropriately applied [10]. In many cases, the process of “trial and error” which does not allow for learning, has been mislabeled as adaptive management. An early review of adaptive management found that it relies excessively on the use of linear systems models, discounts expert knowledge, and does not pay adequate attention to policy processes that promote the development of shared understandings among diverse stakeholders [12]. Indeed, the claim that adaptive management has failed to consider stakeholder values and opinions is a criticism that has shown itself again and again in the literature [3, 12–14]. To be effective, new adaptive management efforts need to incorporate knowledge from multiple sources, make use of multiple systems models, and support new forms of cooperation among stakeholders such as structured decision making [12, 15–16].

Our previous paper proposed an enhanced adaptive management (EAM) approach that integrates structured decision analysis with physical models and monitoring information to provide managers and decision-makers with a framework for understanding how management plans should change based on a given state of knowledge [6]. The EAM approach requires the development of several components:

- A decision framework specifying the criteria to be used in evaluating the remedial alternatives;
- Enumeration of the relative importance or trade-offs among these criteria (relative weights);
- Empirical results and physical models relating the system drivers and changes in the stated criteria;
- Linkages, assumptions, or models to hypothesize the effects of management plans on the system drivers or the project’s evaluation criteria.

In this paper, we have developed a conceptual EAM framework to support remediation of mercury in the South River, near Waynesboro, VA. Mercury was introduced to this area from 1929–1950 when it was used at a former DuPont facility in Waynesboro, VA [17]. This resulted in a fish consumption ban over 105 miles of river that later was reduced to an advisory based on revisions to the Food and Drug Administration’s action level for mercury in fish. In response to the mercury contamination and after careful study and public input, the state selected a Monitored Natural Recovery (MNR) plan and initiated a 100 year monitoring program in 1984. Because mercury levels in tissues of some species of fish were not declining as previously predicted, DuPont and the Virginia Department of Environmental Quality established the South River Science Team in 2001 to understand why fish tissue levels are not decreasing and to evaluate options to address this. In order to maximize likelihood of remedial effectiveness and minimize short-term and long-term risks, a phased adaptive management approach to remediation is under consideration. The remedial objective is to implement an effective remedy that reduces exposure and transport of mercury and that enhances ecological habitat in a cost effective manner. According to the 1990 National Contingency Plan (NCP), a remedy is typically considered cost effective when its cost is proportional to its overall effectiveness [18]. Remedial action is proposed to begin in the first two-mile stretch of the river adjacent to the former plant’s outfall and proceed downstream as necessary.

The approach developed here was designed to be utilized as a screening tool and to support decisions in the context of planning and implementation of South River remediation. Our purpose was to determine if the decision model could be integrated with physical models and assessments that predict the effects of a remedial action, and inform the collection of necessary loading data and monitoring data. Application of this approach allows us to specify the conditions which would change the relative performance of different courses of action. We utilized the published literature from that region to inform the potential effects. Specific Hg concentrations and uncertainties, river flows, loading data, bank management areas (BMAs), and alternative-specific load reduction rates are hypothetical but chosen to reflect current conditions. Demonstration of the model, and evaluation of its utility, can help determine how these measurements can be utilized and the specificity that is necessary in collecting each parameter. Here, a mass-balance approach was implemented to provide an indication of the anticipated change in Hg loading and how uncertainty can be represented in the model and reduced with monitoring. The application was not designed to represent the complexity of Hg loading into the South River, but rather to determine the utility of the adaptive management approach to represent the uncertainty of actions outcomes on remedial goals. Determining if different loading scenarios influence the performance of the different courses of actions can inform if additional measurement or modeling is material to the decision. EAM necessarily requires specification of the criteria along which decisions are made, clear hypotheses about the potential impacts of remedial actions, and suggests a monitoring plan that increases the certainty and accuracy of the remedial outcomes. In this case, the EAM is one of several models that will be linked to provide decision-makers with an increased understanding of the remedy’s effectiveness. The utility and performance of this conceptual model will be assessed in order to determine if EAM will be an asset in remedial selection process.

### Case Application

The approach developed was designed to support phased remedial action in the South River, VA. Concentrations of Hg in the river banks, sediment, and the water column increase downstream of the former DuPont site peaking between relative river mile (RRM) 5–10 for sediment Hg and RRM 10–15 for MeHg in water and in smallmouth bass Results of several studies strongly suggest that the greatest source of Hg into the river is bank erosion especially in the reach from RRM 0, location of the former plant, to RRM 10. Additional sources of Hg to the river include Hg fluxes from deeper sediments to surface sediments, inflows from upstream, floodplain runoff, and to a minor extent, tributaries, groundwater, bank leaching, and residual seepage from the former DuPont plant outfall [19]. Most of the riverbed is categorized as gravel though approximately 15% is made up of fine grained material. The river bed is believed to be a major site of methylation in the river [20], producing MeHg that biomagnifies in the food web and ultimately in human food fish like the smallmouth bass. The relationship between anticipated water column Hg concentration and the concentration of MeHg in smallmouth bass is based on the empirical relationship reported in Brent and Kain, 2011 [21].

## Materials and Methods

### Enhanced Adaptive Management (EAM) Model

**Figure 1:**
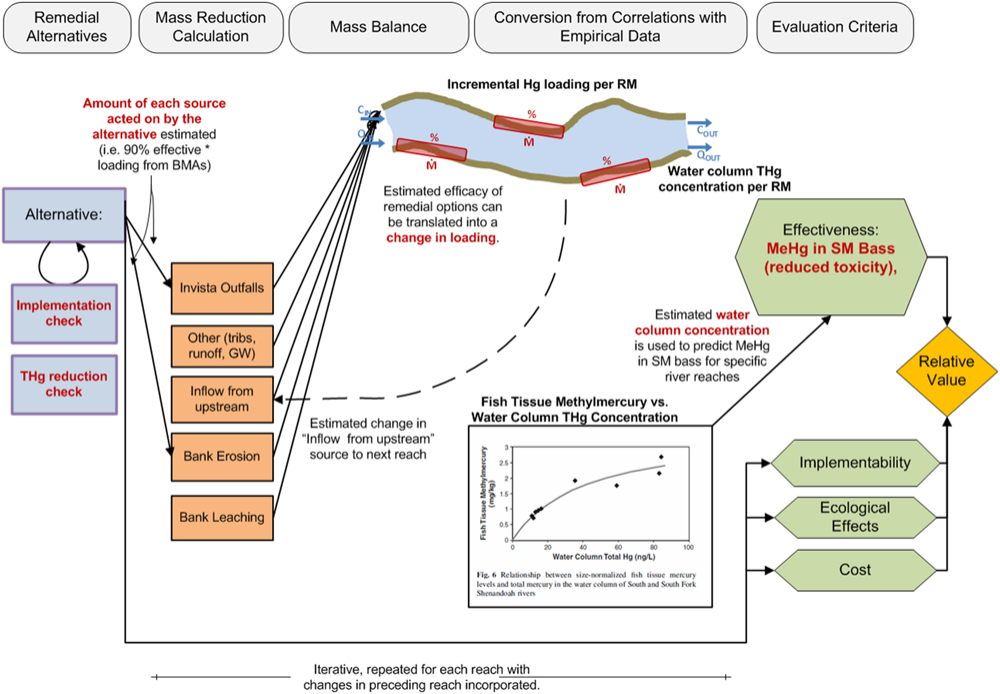
The decision model utilized in the EAM approach for remediation of legacy Hg in the South River. The remedial alternatives need to be specified in terms of their efficiency in reducing the THg loading rate expected in each compartment (orange boxes). This local efficiency in THg loading reduction together with initial river flow rates and water column THg concentrations are utilized in a mass-balance calculation (river schematic) which determines the anticipated change in water column THg loading and concentration in various specified river reaches. The new water column THg concentration is then compared to empirical data (inserted graph) to predict of the smallmouth bass tissue MeHg concentration anticipated at steady state after implementation. The effectiveness of the remedial alternative, as indicated by the anticipated reduction in smallmouth bass MeHg, is combined with the implementability, ecological effects and estimated cost of that alternative to calculate the relative value associated with that specific remedial approach.

EAM requires the development of a quantitative decision model that links the estimated effects of remedial actions with the criteria for a successful project or management plan. The evaluation criteria, as well as the metrics used to inform those criteria, should be developed by a diverse and comprehensive stakeholder group. In this case the decision model is a deterministic, multi-attribute model (Fig. 1) that calculates the relative value or utility of different courses of action according to the performance criteria. The uncertainty in the data is specified, and simulation is used to develop probabilistic characterization of performance. One attribute, the effectiveness in the reduction of smallmouth bass tissue MeHg, is predicted by a mass balance model which utilizes the specifics of each remedial action alternative to determine potential changes in water column Hg concentration.

### Decision Model

The decision model was designed to reflect the decision process for ranking the remedial alternatives based on the expected effects of their implementation. The evaluation criteria are considered to be (1) the effectiveness as measured by the relative, anticipated effectiveness in reduction of MeHg in smallmouth bass tissue, (2) the ecological effects as reflected in two metrics, the potential of an alternative to create habitat and to avoid risks to the ecological community, (3) the implementability as reflected in two metrics, the constructability and relative land owner support, and (4) the cost of implementation plus 20 years of maintenance. Additional physical models predicting changes in Hg flux, specific species risk, and other measures of implementability could be integrated as available and predictive of the anticipated response to remedial actions. A relative risk model for any ecological receptor of interest can be included as a measurement of the probability of an action to result in ecological disruption. For this demonstration, categorical judgments are included as indicators of the ecological effects reflecting a current understanding of the relative performance of remedial alternatives. The relative importance of each of these criteria is captured in a series of weights; an initial weighting scheme was set at Effectiveness—40%, Ecological Effects—25%, Implementability—25% and Cost—10%.

**Figure 2:**
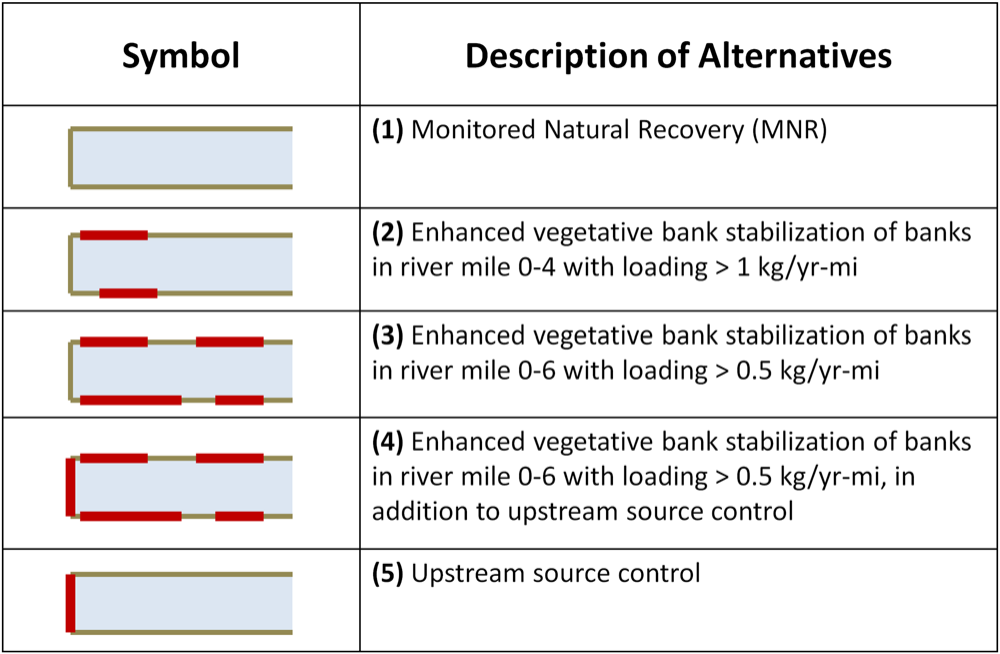
Remedial alternatives considered in the case study. The alternatives are different combinations of measures including vegetative bank stabilization, monitored natural recovery (MNR) in the reaches closest to the outfall, and outflow source control.

A set of remedial alternatives were developed for the case study (Fig. 2). The alternatives are different combinations of measures including vegetative bank stabilization, monitored natural recovery (MNR) in the reaches closest to the outfall, and outflow source control. The performance of each alternative was calculated based on each of the 6 metrics comprising the 4 evaluation criteria. The effectiveness criterion reflecting the anticipated average concentration of MeHg in smallmouth bass 20 years post alternative implementation was calculated using a mass balance model and the empirical relationship between water column total Hg (THg) concentration and smallmouth bass tissue MeHg concentration reported in Brent and Kain [21]. The performance of each alternative on the metrics comprising the implementability and ecological effects criteria were specified on a high-medium-low (1, 0.5, 0) scale based on expert judgment and are reported in Table 1. A range of probable costs for each alternative was also specified based on the mean, minimum, and maximum probable cost of implementing and maintaining each measure. Costs are included in the model only using current value. These initial parameters would be expected to be updated utilizing study and monitoring data collected following implementation. Monitoring and implementation outcomes are used to revise and recalculate the model, reducing uncertainty and changing the relative performance of different alternatives.

**Table 1:** Initial performance of each remedial alternative on the evaluation criteria.

**Aspect**	**Description**	**Alternative Score 1 2 3 4 5**				
**EFFECTIVENESS**						
Efficacy of remedial action	For this example, the vegetative stabilization of banks was assumed to be 90% effective and monitored natural recovery is assumed to be 2% effective. Outflow reduction from upstream source control was calculated to reduce the entering Hg concentration upstream of river mile 0 to 0 ng/L.					
**IMPLEMENTABILITY**						
Performance Risk	The risk of mechanical failure of remediation measures is estimated along the range of low (0) to high (1) according the type of stabilization and its extent.	0	0.5	0.5	0.5	0
Land Owner Approval	Support for remediation projects is estimated on the range of low (0) to high (1) according to level and extent of the disruption anticipated the anticipated efficacy, and the number of landowners that will be involved.	1	0.5	0.25	0	0.25
**ECOLOGICAL EFFECTS**						
Benefits—Habitat creation	The potential for habitat creation was ranked for each alternative from low (0) to high (1) according the length of bank modification.	0	0.25	1	1	0
Risk—Ecological function, community	Risk to ecological function from implementation from each alternative was ranked as high (1) to low (0) according to the extent of the disruption along the river bank.	0	0.25	1	1	0
**COST**						
Implementation and Maintenance Cost	The range of costs (minimum to maximum) is estimated according to the type and amount of each remedial alternative to implemented.	0	$5–10M	$20–40M	$60–160M	$40–120M

### Effectiveness—Mass Balance

The mass balance model utilizes the concentrations and flow rates in and out of the specific reaches of the river, the anticipated efficacy of remedial alternatives, and an empirical relationship between water column THg and smallmouth bass tissue MeHg derived by Brent and Kain [21] for the South River. From these inputs, calculations are utilized to predict loading patterns, water column Hg, and smallmouth bass MeHg in each reach once the concentrations return to a steady state following the impacts of the remedial actions. The model contains no temporal component. Rather, it calculates the change in Hg once the system reaches a steady state. Adaptive management depends on monitoring and updating the uncertain parameters following implementation of a remedial action. The reduction in Hg loading from a specified BMA following remedial action implementation was initially set at 90% after 3 years, 2% after 3 years and 100% immediately for vegetative bank stabilization, MNR and outflow source control, respectively. Otherwise, the flow of Hg in the system is calculated from empirical data. The initial efficacies of the interventions are based on expert judgment, pilot studies and monitoring results. Many of the parameters of the model incorporate uncertainty, specifically water column Hg concentration, individual BMA loading rates and anticipated costs. For the initial calculations, river flow rates and water column Hg concentrations were included as point values, while the uncertainty in Hg unit loading to the river from erosion at each BMA was captured through the use of a triangular function. Each triangular function has three inputs- the minimum possible value, the most likely value, and the maximum possible value.

To calculate the effectiveness of each remedial alternative, the river was first split into five reaches, each two miles in length starting with RRM 0 at the original point source of mercury contamination and ending at RRM 10. The mass balance equation was then applied to each reach to calculate the initial mass rate of Hg entering the water column in the reach annually, or the initial external Hg loading, *Ṁ_in,ext(t0)_*,

$${\dot{M}}_{in,ext(t}{}_{0)}=\;C_{out\;(t}{}_{0)}\cdot Q_{out}-\;C_{in(t}{}_{0)}\cdot Q_{in}.$$ *Equation* 1

*C_out_* and *C_in_* represent the averaged concentration in the river stream segment measured from the water column entering and exiting the reach annually; *Q_out_* and *Q_in_* represent the volumetric flux of water entering and exiting the reach annually; *t_0_* indicates the time prior to implementation of any remedial action. Use of this approach assumes the water in the reach is fully mixed such that the water column concentration of Hg exiting the reach, *C_out_* is equal to the water column concentration of Hg within the reach. Average annual run off before and after implementation should reflect the change in Hg flux despite weather related or seasonally induced changes.

Once the initial loading is known, the new loading after the alternative is implemented and once the system reaches a new steady state water column concentration (*t = t_1_*), is calculated as,

$${\dot{M}}_{in,ext(t}{}_{1)}=\;{\dot{M}}_{in,ext(t0)}-\;\Delta {\dot{M}}_{in,ext(}{}_{t1-t0)},$$ *Equation* 2

where *ΔṀ_in,ext(t1-t0)_* is the change in the rate of Hg mass entering the water column in the reach from all local sources external to the water column. To calculate this change in loading, the left and right banks of each river reach were further divided into individual 0.2 mile-long bank management areas (BMAs) each of which has its own average Hg soil concentration and annual Hg unit loading rate. Remedial measures were then hypothetically applied to the river, to specific reaches, and to any of the individual BMAs specified for each alternative (S1 Table). The expected change in annual Hg unit loading to each reach (kg Hg/yr-mile), *ΔṀ_in,ext(t1-t0)_*, was calculated for each of the five remedial alternatives based on the sum of the initial Hg unit loading in each BMA, and the anticipated percent reduction in that loading expected from implementing the remedial alternatives.

Next the mass balance equation (Equation 1) is rearranged to solve for the new concentration of Hg in the water column within and exiting each reach at the new steady state, *C_out(t1)_ = (C_in(t1)_ · Q_in_ + Ṁ_in,ext(t1)_)/Q_out_*. Finally, the new MeHg concentration in smallmouth bass tissue is calculated using the empirical equation derived by Brent and Kain [21],

$$Hg_{Fish}=\;1/(11.682\;\cdot \;Hg_{Water}+\;0.282).$$ [21]

*Hg_Fish_* is the size normalized smallmouth bass fish tissue MeHg concentration; *Hg_Water_* is the concentration of THg in the water column (written as *C_out(t1)_* above). In this case, the smallmouth bass is assumed to have a home range equal to RRM0-RRM10 so that the value of each alternative’s effectiveness performance criterion is the calculated *Hg_Fish_* value averaged over the five reaches.

### Prioritization of Remedial Alternatives

The model uses the calculated MeHg reduction in smallmouth bass and the performance of the alternatives on each metric to calculate the relative value of each alternative. Multi-attribute value theory is used to compare the alternatives using a local scale for each metric [22]. The “value” of each alternative is a normalized score for each metric with the highest performing alternative(s) given a value score of 1, and the lowest performing alternative(s) given a 0. The total utility, *U*(***a***), for an alternative, ***a***, is calculated as a weighted sum across the four criteria,

$$U\left(a\right)\;=\;w_{1}\cdot \;V_{1}\left(a_{1}\right)\;+\;\ldots \;+\;w_{n}\cdot \;V_{n}\left(a_{n}\right),$$ [23]

where *a_i_* is the performance score of alternative ***a*** on criterion *O_i_* for *i* = 1 to *n* with *n* = the number of criteria, *V_i_(a_i_)* is the value of alternative ***a*** reflecting its performance on criterion *O_i_* and *w_i_* is the weight of criterion *O_i_* where Σ*w_i_* = 1. To capture the uncertainty in the anticipated efficacy and cost estimation, two additional calculations were performed. The possible extent of the performance was calculated utilizing the minimum and maximum probable values for efficacy and cost for each alternative. In addition, a Monte Carlo simulation was performed (1000 iterations) bounded by the triangle probability function specified by the mean, minimum, and maximum values for each measure.

### Monitoring after Implementation

The model is designed to reflect an understanding of the current conditions within the river. Another output of the model, therefore, is a series of measurements that should be taken in order to update the model, reduce uncertainty and increase understanding of the relationship between parameters that influence the predicted outcomes. The parameters form the basis of a short- and long-term monitoring plan which is necessary to inform the ranking of alternatives in the second phase of implementation.

### Sensitivity Analysis

Sensitivity analysis was used to provide insight into the effects of differences in priorities, as reflected in the weights on criteria, on the relative performance of alternatives. The initial weighting scheme was set at Effectiveness—40%, Ecological Effects—25%, Implementability—25% and Cost—10%. Three alternative weighting schemes were considered as part of a sensitivity analysis. One in which 100% of the criteria weight was placed on the effectiveness of the alternative in reduction in smallmouth bass MeHg concentration. A second in which 50% of the criteria weight was effectiveness and 50% was ecological effects. A third set of weights specified that 40% of the criteria weight was determined by the effectiveness calculation, 30% on implementability and 30% on cost.

## Results and Discussion

### Performance of Remedial Alternatives

**Figure 3:**
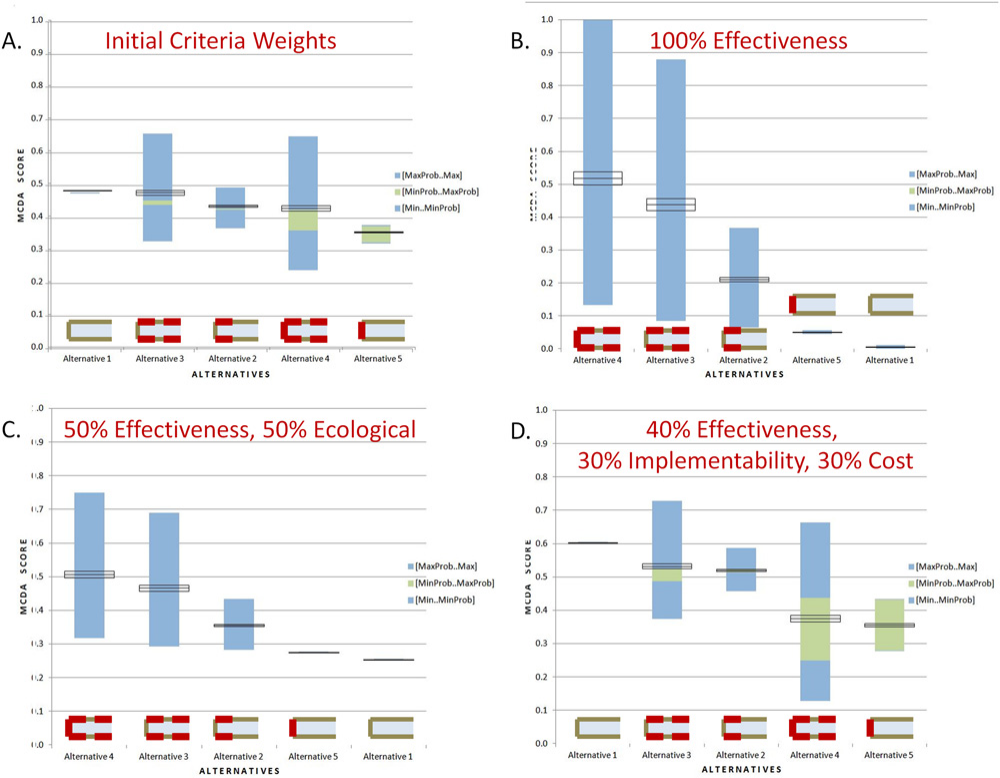
A basic sensitivity analysis of the effect of the criteria weights on the performance. The remedial alternatives are ranked according to their mean value score. For each alternative, black boxes indicate the mean and standard deviation of 1000 simulations across the range of probable values. The green box shows the highest and lowest possible scores achieved with the maximum and minimum probable values for that alternative, respectively. The blue boxes show the range of scores between the probable value and the possible value for each alternative; above the mean is the range of scores between the maximum probable and the maximum possible while the minimum range is below. (A.) The original weighting scheme of 40% effectiveness, 25% ecological effect, 25% implementability and 10% cost results in Alternatives 1 and 3 providing the highest. (B.) For this calculation, 100% of the criteria weight was placed on the effectiveness of the alternative in reduction in smallmouth bass MeHg concentration. The most aggressive alternative, Alternative 4 which includes both upstream sources control and extensive bank stabilization, has the highest mean value. (C.) For this calculation, 50% of the criteria weight was effectiveness and 50% was ecological effects. Again, Alternative 4 has the highest mean value, and the reduction in uncertain associated with the inclusion of ecological effect allows it to outperform Alternatives 4 and 5. (D.) The case with 40% of the criteria weight on effectiveness, 30% on implementability and 30% on cost also results in Alternatives 1 and 4 outperforming the other alternatives.

The model generates a range of scores reflecting the value and uncertainty associated with each of the five remedial alternatives under the current conditions and the current understanding of the system. The evaluation of the current case resulted in the relative ranking of the alternatives shown in Fig. 3A. The highest performing alternatives are alternative 1 and alternative 3 with mean value scores of 0.477 and 0.471 respectively. Their performance is followed closely by alternatives 2 and 4 with mean value scores of 0.430 and 0.424, respectively. Alternative 4 has relatively high uncertainty; the probable range of value scores for alternative 4 is 0.362–0.425. Alternative 5 scored the lowest and has a noticeable range of probable values, with an average of 0.351 and a probable range of 0.325 to 0.376. Alternative 1 (MNR) has the least uncertainty because of the low rate of Hg reduction (2%) and the small range of potential costs for this alternative. The upstream outflow control adds uncertainty to the performance of Alternative 4 and 5 because of the range in the estimated cost of complete reduction in source control. This uncertainty reduces the mean performance of those alternatives that include outflow control. Though the possible value scores for most of the alternatives overlap because of uncertainty in cost and unit Hg loading rates, the probable ranges reflect somewhat more conclusive rankings.

### Water column THg and smallmouth bass MeHg

**Figure 4:**
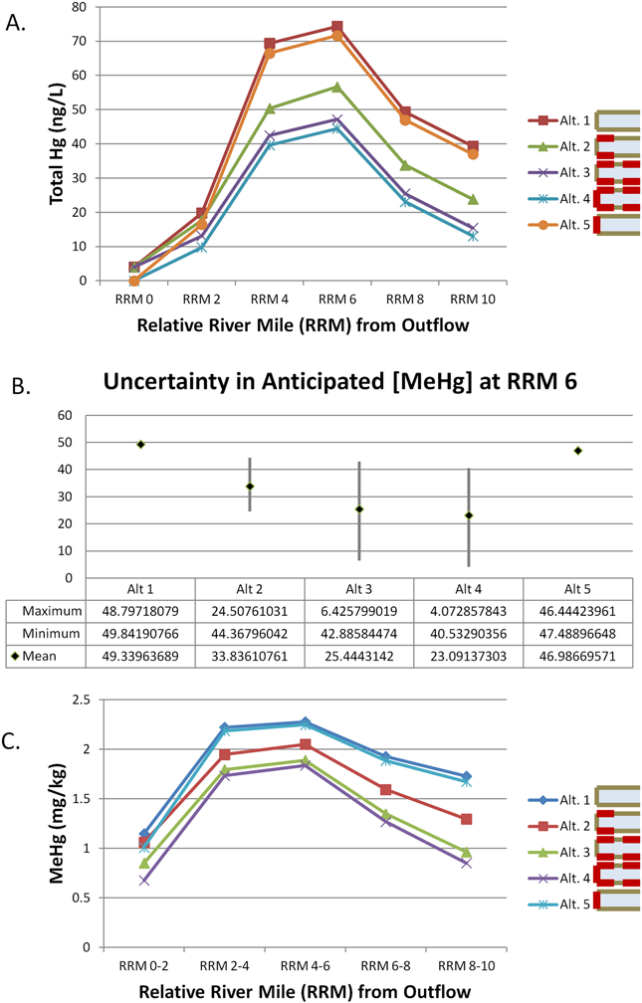
THg concentration predictions from the mass-balance model for the initial alternatives. (A.) The mean predicted water column concentrations in ng/L are reported for each alternative for each reach of the river as calculated by the mass balance model. Alternatives 1 and 5 result in higher predicted THg concentrations then the other alternatives along the length of the river. (B.) The uncertainty in anticipated water column THg concentration at relative river mile 6 is shown as predicted by the mass balance model. Below the graphical display are the calculated values for the maximum, minimum and mean concentration of THg (ng/L). Alternatives 1 and 5 are anticipated to result in higher mercury levels in this stretch of the river. The other alternatives cannot be distinguished because of the uncertainty in the loading multiplied by the anticipated rate of reduction. (C.) The mean predicted concentrations of MeHg in smallmouth bass tissue (mg/kg) are reported for each alternative in each river reach as calculated by the mass balance model. Following from the water concentrations, Alternatives 1 and 5 result in higher fish tissue concentrations than the other options.

The mass balance module predicts changes in water column THg following alternative implementation after the expected time to reach a steady state has passed. The estimated mean water column THg concentrations in each reach for each alternative are shown in Fig. 4A. Alternatives 3 and 4, which include the most extensive bank stabilization, result in the lowest mean water column total Hg concentrations after RRM 2 followed by alternative 2 with less extensive bank stabilization. Finally alternatives 1 and 5, those not involving any bank stabilization, are predicted to result in the highest mean total Hg concentrations in the water column. Again, uncertainty in the annual unit Hg loading at each BMA results in a large range of possible water column total Hg concentrations for alternatives 2, 3 and 4. At river mile 8, this uncertainty results in no significant difference between the bank stabilization alternatives, though MNR and upstream source control alone have significantly higher total Hg concentrations (Fig. 4). The anticipated MeHg concentration in smallmouth bass tissue is predicted based on the predicted water column total Hg concentrations derived from the mass balance model. Mean predicted concentrations are shown in Fig. 4C for each alternative.

### Criteria weights

The model assumptions include an assumption that the criteria have different levels of importance, and therefore, different weights were placed on the four criteria—effectiveness, ecological effects, implementability and cost (Fig. 3). If 100% of the weight is placed on the effectiveness in reduction of MeHg in smallmouth bass, Alternative 4, the most aggressive alternative, has the highest mean value of all the alternatives (Fig. 3B). However, the uncertainty associated with effectiveness in this alternative stretched the performance such that its range of possible value overlaps with the other bank stabilization inclusive alternatives, 2 and 3. A third weighting scheme placed half of the weight on effectiveness and half on ecological effects. Under this condition, Alternative 4 retains the highest value. Emphasizing ecological effects reduces the uncertainty to a level that Alternative 4 completely outperforms Alternatives 1 and 5 (Fig. 3C). Considering only effectiveness (40%), implementability (30%) and cost (30%), does not change the relative performance of the 5 alternatives in comparison with the initial assumptions (Fig. 3D and 3A). However, removing the emphasis on ecological effect and increasing the emphasis on cost increases the utility score on all alternatives. As well, those alternatives that include source control at the outflow (4 and 5) have a larger range of probable utility reflecting the uncertainty in the cost of those alternatives.

### Monitoring Plan

The EAM approach includes the production of a list of parameters that should be monitored in the short-term and in the long-term to update the model, reduce uncertainty and learn from initial implementation efforts. In this case study, the parameters in the model that would need to be monitored at the reach level would be the concentration of THg in the water column and corresponding flow rate; these should be monitored as each implementation is expected to reach equilibration (short-term). To monitor the impacts of vegetative bank stabilization, individual BMAs should be assessed in the short-term for changes in Hg unit loading through measurements of bank stability and Hg uptake by biota adjacent to the bank, for ecological health and habitat enhancement through ecological metrics, and for land owner acceptance. Following the initial implementation, these data should be utilized to update the decision model before future actions are considered. Long-term monitoring should include THg in the water column and MeHg in smallmouth bass. These data should be used to update the relationships in the decision model and determine to what extent the evaluation criteria of the remedial action are being met with the current approach. Completion of this process in the first two-mile reach of the river provides a foundation for learning following implementation, which is valuable as remediation efforts progress further downstream.

## Conclusions

The EAM approach is designed to assist and focus decision-making under uncertainty about remedial performance and limited understanding of the system responses. EAM includes a decision model which serves as both a record of the understanding of the system as it relates to the decision criteria, and a way to compare different courses of action. A mass-balance approach provided an example of how physical models can be embedded to inform the uncertainty and potential reduction in Hg loading. The mass-balance by river reach was not designed to represent the complexity of Hg loading into the South River, but rather to determine the utility of inclusion of formalized decision modeling in a structured decision making approach. The importance of measuring or modeling specific loading from BMA is proportional to the importance of the “effectiveness” criterion in the decision model. As an example of its first function, the model predicts MeHg concentration in smallmouth bass once a new steady state is reached. It does not provide a mechanism or a prediction of changes in the distribution of Hg in the system, but it provides a simple description of the relationship between actions (remedial alternatives) and their impacts on the evaluation criteria (reduction in MeHg in smallmouth bass, implementability, etc.). The decision model forces a quantitative evaluation of alternatives and a relative value score is calculated reflecting how well the alterative meets all of the criteria. Therefore, the performance of each remedial alternative can be compared and the evaluation provides information to the remedial action team on additional information or clarification that would further distinguish the alternatives.

The other critical aspects of any adaptive management approach are monitoring, evaluation, learning and adjustment. Following implementation of any action, a series of short- and long-term endpoints need to be monitored and the process analyzed for any lessons to be learned. These measurements, and their implications, should be incorporated into any future decisions. The EAM approach allows for inclusion of the new monitoring results into the model, reducing uncertainty and allowing for a new assessment of the system. Therefore the learning and adjustment phases of the process are simplified by allowing circulation of an updated decision model and a re-analysis of new alternatives given the increased understanding of the system. EAM should focus the remedial implementation team on collecting those data that are material to the decision, and those factors that allow better predictions of the behavior of the system. The applications can be used to prioritize the collection or development of data on the loading of Hg according to the importance in informing the relative performance of alternative courses of action. It also should restrict discussion of future actions through previous specification and quantification of the decision criteria; these are not expected to change significantly over the life of the project.

Development of a case study for the South River, VA provides insight into the type and quality of outcomes anticipated using the EAM approach (Fig. 3). These results are the type of details that should be considered by a remedial action team in advance of their decision to proceed in a specific course of action. They indicate that, if the remedial action team places a high value on the reduction in smallmouth MeHg, then further consideration should be given to extensive bank stabilization. However, if implementability and cost are the main concerns, then MNR or more limited bank stabilization is a course of action that should be considered. Uncertainty in bank loading and costs result in large, often overlapping, ranges of possible utility scores for the alternatives. Reducing these uncertainties could change the relative rankings of the alternatives. The distribution of weights among criteria influences the relative ranking of alternatives. Application of the model to the available data in the South River may produce similar outcomes. As the tool is calibrated to reflect the understanding and priorities of the team during the actions in the first reach of the river, it becomes more useful in processing future monitoring results and specification of thresholds for switching between alternative actions.

### Divergence from traditional adaptive management approaches

**Figure 5:**
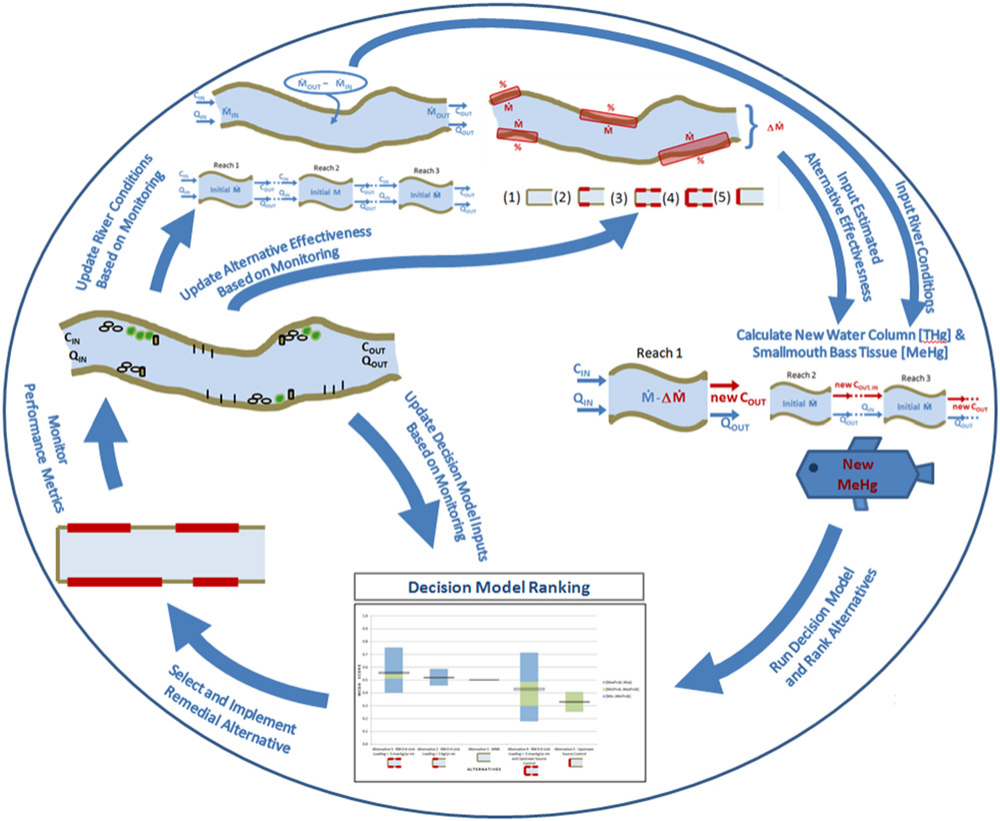
The process for EAM as demonstrated by the case study. Beginning at the top, the river conditions and change in total Hg loading in response to each alternative are estimated based on monitoring and pilot study results. These are then input into the mass balance to calculate the water column total Hg concentration and the smallmouth bass tissue MeHg concentration both initially and following alternative implementation. The smallmouth bass tissue concentrations are then entered into the decision model along with performance scores for the other criteria and preference weights and a relative ranking of alternatives is calculated. Based on the outcome of the decision model, an alternative is selected and implemented. Metrics that relate to parameters in the decision model are monitored according to the monitoring plan and updated as necessary. The process then repeats with the latest information gathered through monitoring and research.

The EAM approach to adaptive management outlined here differs from traditional approaches to adaptive management in several significant ways (Fig. 5) [6]. Development and choice of a remediation course of action under this approach begins with development of a structured decision model for a specific project. The remedy evaluation criteria must be agreed upon in advance as well as the relative importance of these criteria in the form of weights. In order to implement this tool in the South River remediation context, a substantial effort would have to be made not only to clarify evaluation criteria and weights but also to incorporate specific metrics for ecological effects and implementability. Additional results of pilot studies, physical models and other data may be incorporated into the conceptual system model and mass balance approach to link characteristics and drivers with the management objectives and reduce the initial uncertainty. For example, an integrated regional risk model utilizing different species as ecological receptors would develop quantitative risk evaluations which could replace the judgment currently included as a comparison of the ecological risk of different actions. Implementation of EAM requires more problem framing and data analysis as the process is begun than traditional adaptive management.

### Benefits of this approach

There are several reasons for utilizing EAM. The approach ensures that: (1) Clear links are established between management choices and current understanding, monitoring information, and project evaluation criteria; (2) Delays resulting from debate about adaptation to monitoring results are reduced; (3) Monitoring plans can be evaluated based on the contribution to improving the decision model; (4) Remedial plans can be updated with new information. Perhaps the most important benefit of the approach is that it quantitatively links decisions to technical understanding of the system to be managed and monitoring information. Analysis and refinement of these linkages provides an opportunity to learn more about the functioning of the ecosystem, to update the projected outcomes of alternative approaches, and to change the model as conditions change in the system.

## Supporting Information

S1 Table Loading for each BMA as conceptualized in the mass balance model. (DOCX)
