# Supplementary material for: An Enhanced Adaptive Management Approach for Remediation of Legacy Mercury in the South River
Source: PLoS One. 2015 Feb 9;10(2):e0117140. doi: 10.1371/journal.pone.0117140 (PMC4321986; doi:10.1371/journal.pone.0117140)
Supplement: S1 Table — (DOCX) [file pone.0117140.s001.docx]

| Reach (Control  Volume) | BMA | RRM Start | RRM End | BMA miles | Min | Min Likely | Mean | Max  Likely | Max |
| --- | --- | --- | --- | --- | --- | --- | --- | --- | --- |
| RM 0‐2 | L1 | 0 | 0.2 | 0.2 | 0 | 0.099614 | 0.09961 | 0.099614 | 0.25 |
|  | L2 | 0.2 | 0.4 | 0.2 | 0 | 0.074914 | 0.07491 | 0.074914 | 0.25 |
|  | L3 | 0.4 | 0.6 | 0.2 | 0 | 0.21682 | 0.21682 | 0.21682 | 0.25 |
|  | L4 | 0.6 | 0.8 | 0.2 | 0 | 0.056645 | 0.05665 | 0.056645 | 0.25 |
|  | L5 | 0.8 | 1 | 0.2 | 0 | 0.083493 | 0.08349 | 0.083493 | 0.25 |
|  | L6 | 1 | 1.2 | 0.2 | 0 | 0.267942 | 0.26794 | 0.267942 | 0.5 |
|  | L7 | 1.2 | 1.4 | 0.2 | 0 | 0.143967 | 0.14397 | 0.143967 | 0.5 |
|  | L8 | 1.4 | 1.6 | 0.2 | 0 | 0.061004 | 0.06100 | 0.061004 | 0.5 |
|  | L9 | 1.6 | 1.8 | 0.2 | 0 | 0.089548 | 0.08955 | 0.089548 | 0.5 |
|  | L10 | 1.8 | 2 | 0.2 | 0 | 0.383328 | 0.38333 | 0.383328 | 0.5 |
|  | R1 | 0 | 0.2 | 0.2 | 0 | 0.092789 | 0.09279 | 0.092789 | 0.25 |
|  | R2 | 0.2 | 0.4 | 0.2 | 0 | 0.031239 | 0.03124 | 0.031239 | 0.25 |
|  | R3 | 0.4 | 0.6 | 0.2 | 0 | 0.162229 | 0.16223 | 0.162229 | 0.25 |
|  | R4 | 0.6 | 0.8 | 0.2 | 0 | 0.014626 | 0.01463 | 0.014626 | 0.5 |
|  | R5 | 0.8 | 1 | 0.2 | 0 | 0.150178 | 0.15018 | 0.150178 | 0.5 |
|  | R6 | 1 | 1.2 | 0.2 | 0 | 0.315793 | 0.31579 | 0.315793 | 0.5 |
|  | R7 | 1.2 | 1.4 | 0.2 | 0 | 0.460448 | 0.46045 | 0.460448 | 0.5 |
|  | R8 | 1.4 | 1.6 | 0.2 | 0.5 | 0.695641 | 0.69564 | 0.695641 | 1 |
|  | R9 | 1.6 | 1.8 | 0.2 | 0.5 | 0.5 | 0.50000 | 0.5 | 0.5 |
|  | R10 | 1.8 | 2 | 0.2 | 0.25 | 0.87877 | 0.87877 | 0.87877 | 1 |
|  | Total |  |  | 4 | 0.0625 | 0.238949 | 0.238949 | 0.238949 | 0.45 |
| RM 2‐4 | L1 | 2 | 2.2 | 0.2 | 0 | 0.227555 | 0.22755 | 0.227555 | 0.5 |
|  | L2 | 2.2 | 2.4 | 0.2 | 0 | 0.044258 | 0.04426 | 0.044258 | 0.5 |
|  | L3 | 2.4 | 2.6 | 0.2 | 0 | 0.216623 | 0.21662 | 0.216623 | 0.5 |
|  | L4 | 2.6 | 2.8 | 0.2 | 0 | 0.081754 | 0.08175 | 0.081754 | 0.5 |
|  | L5 | 2.8 | 3 | 0.2 | 0.5 | 1.141238 | 1.14124 | 1.141238 | 2.5 |
|  | L6 | 3 | 3.2 | 0.2 | 0 | 0.189522 | 0.18952 | 0.189522 | 0.5 |
|  | L7 | 3.2 | 3.4 | 0.2 | 0.5 | 1.93827 | 1.93827 | 1.93827 | 2 |
|  | L8 | 3.4 | 3.6 | 0.2 | 0.5 | 1.021309 | 1.02131 | 1.021309 | 1.5 |
|  | L9 | 3.6 | 3.8 | 0.2 | 0 | 0.41636 | 0.41636 | 0.41636 | 0.5 |
|  | L10 | 3.8 | 4 | 0.2 | 0 | 0.231677 | 0.23168 | 0.231677 | 0.5 |
|  | R1 | 2 | 2.2 | 0.2 | 0 | 0.207022 | 0.20702 | 0.207022 | 0.5 |
|  | R2 | 2.2 | 2.4 | 0.2 | 0 | 0.376954 | 0.37695 | 0.376954 | 0.5 |
|  | R3 | 2.4 | 2.6 | 0.2 | 0 | 0.002386 | 0.00239 | 0.002386 | 0.5 |
|  | R4 | 2.6 | 2.8 | 0.2 | 0 | 0.196766 | 0.19677 | 0.196766 | 0.5 |
|  | R5 | 2.8 | 3 | 0.2 | 0 | 0.049211 | 0.04921 | 0.049211 | 0.5 |
|  | R6 | 3 | 3.2 | 0.2 | 0.5 | 1.364609 | 1.36461 | 1.364609 | 1.5 |
|  | R7 | 3.2 | 3.4 | 0.2 | 0.5 | 3.10302 | 3.10302 | 3.10302 | 3.5 |
|  | R8 | 3.4 | 3.6 | 0.2 | 0.5 | 0.952435 | 0.95243 | 0.952435 | 3.5 |
|  | R9 | 3.6 | 3.8 | 0.2 | 0.5 | 3.252688 | 3.25269 | 3.252688 | 3.5 |
|  | R10 | 3.8 | 4 | 0.2 | 0.5 | 1.136567 | 1.13657 | 1.136567 | 1.5 |
|  | Total |  |  | 4 | 0.2 | 0.807511 | 0.807511 | 0.807511 | 1.275 |
|  | L1 | 4 | 4.2 | 0.2 | 0 | 0.359567 | 0.35957 | 0.359567 | 0.5 |
|  | L2 | 4.2 | 4.4 | 0.2 | 0 | 0.233699 | 0.23370 | 0.233699 | 0.5 |
|  | L3 | 4.4 | 4.6 | 0.2 | 0 | 0.168624 | 0.16862 | 0.168624 | 0.2 |
|  | L4 | 4.6 | 4.8 | 0.2 | 0.5 | 0.676454 | 0.67645 | 0.676454 | 1 |

| Reach (Control  Volume) | BMA | RRM Start | RRM End | BMA miles | Min | Min Likely | Mean | Max  Likely | Max |
| --- | --- | --- | --- | --- | --- | --- | --- | --- | --- |
| RM 4‐6 | L5 | 4.8 | 5 | 0.2 | 0 | 0.080871 | 0.08087 | 0.080871 | 0.2 |
|  | L6 | 5 | 5.2 | 0.2 | 0 | 0.255947 | 0.25595 | 0.255947 | 0.5 |
|  | L7 | 5.2 | 5.4 | 0.2 | 0.25 | 0.63661 | 0.63661 | 0.63661 | 1 |
|  | L8 | 5.4 | 5.6 | 0.2 | 0 | 0.181656 | 0.18166 | 0.181656 | 0.2 |
|  | L9 | 5.6 | 5.8 | 0.2 | 0 | 0.00095 | 0.00095 | 0.00095 | 0.2 |
|  | L10 | 5.8 | 6 | 0.2 | 0 | 0.575765 | 0.57576 | 0.575765 | 0.75 |
|  | R1 | 4 | 4.2 | 0.2 | 0 | 0.074587 | 0.07459 | 0.074587 | 0.2 |
|  | R2 | 4.2 | 4.4 | 0.2 | 0 | 0.098966 | 0.09897 | 0.098966 | 0.2 |
|  | R3 | 4.4 | 4.6 | 0.2 | 0 | 0.031597 | 0.03160 | 0.031597 | 0.2 |
|  | R4 | 4.6 | 4.8 | 0.2 | 0 | 0.075985 | 0.07599 | 0.075985 | 0.2 |
|  | R5 | 4.8 | 5 | 0.2 | 0 | 0.032651 | 0.03265 | 0.032651 | 0.2 |
|  | R6 | 5 | 5.2 | 0.2 | 0 | 0.013438 | 0.01344 | 0.013438 | 0.2 |
|  | R7 | 5.2 | 5.4 | 0.2 | 0 | 0.079168 | 0.07917 | 0.079168 | 0.2 |
|  | R8 | 5.4 | 5.6 | 0.2 | 0 | 0.085852 | 0.08585 | 0.085852 | 0.2 |
|  | R9 | 5.6 | 5.8 | 0.2 | 0 | 0.079614 | 0.07961 | 0.079614 | 0.2 |
|  | R10 | 5.8 | 6 | 0.2 | 0 | 0.103353 | 0.10335 | 0.103353 | 0.2 |
|  | Total |  |  | 4 | 0.0375 | 0.192268 | 0.192268 | 0.192268 | 0.3525 |
| RM 6‐8 | L1 | 6 | 6.2 | 0.2 | 0 | 0.007393 | 0.00739 | 0.007393 | 0.2 |
|  | L2 | 6.2 | 6.4 | 0.2 | 0 | 0.180826 | 0.18083 | 0.180826 | 0.2 |
|  | L3 | 6.4 | 6.6 | 0.2 | 0 | 0.697881 | 0.69788 | 0.697881 | 1 |
|  | L4 | 6.6 | 6.8 | 0.2 | 0 | 0.083074 | 0.08307 | 0.083074 | 0.1 |
|  | L5 | 6.8 | 7 | 0.2 | 0 | 0.277686 | 0.27769 | 0.277686 | 0.5 |
|  | L6 | 7 | 7.2 | 0.2 | 0 | 0.048496 | 0.04850 | 0.048496 | 0.1 |
|  | L7 | 7.2 | 7.4 | 0.2 | 0 | 0.072898 | 0.07290 | 0.072898 | 0.1 |
|  | L8 | 7.4 | 7.6 | 0.2 | 0 | 0.071081 | 0.07108 | 0.071081 | 0.1 |
|  | L9 | 7.6 | 7.8 | 0.2 | 0 | 0.08807 | 0.08807 | 0.08807 | 0.1 |
|  | L10 | 7.8 | 8 | 0.2 | 0 | 0.043225 | 0.04322 | 0.043225 | 0.2 |
|  | R1 | 6 | 6.2 | 0.2 | 0 | 0.199893 | 0.19989 | 0.199893 | 0.2 |
|  | R2 | 6.2 | 6.4 | 0.2 | 0 | 0.066258 | 0.06626 | 0.066258 | 0.5 |
|  | R3 | 6.4 | 6.6 | 0.2 | 0 | 0.068551 | 0.06855 | 0.068551 | 0.1 |
|  | R4 | 6.6 | 6.8 | 0.2 | 0 | 0.018573 | 0.01857 | 0.018573 | 0.1 |
|  | R5 | 6.8 | 7 | 0.2 | 0 | 0.008127 | 0.00813 | 0.008127 | 0.1 |
|  | R6 | 7 | 7.2 | 0.2 | 0 | 0.03889 | 0.03889 | 0.03889 | 0.1 |
|  | R7 | 7.2 | 7.4 | 0.2 | 0 | 0.044855 | 0.04485 | 0.044855 | 0.1 |
|  | R8 | 7.4 | 7.6 | 0.2 | 0 | 0.056315 | 0.05632 | 0.056315 | 0.1 |
|  | R9 | 7.6 | 7.8 | 0.2 | 0 | 0.084614 | 0.08461 | 0.084614 | 0.1 |
|  | R10 | 7.8 | 8 | 0.2 | 0 | 0.007577 | 0.00758 | 0.007577 | 0.1 |
|  | Total |  |  | 4 | 0 | 0.108214 | 0.108214 | 0.108214 | 0.205 |
|  | L1 | 4 | 4.2 | 0.2 | 0 | 0 | 0.00000 | 0 | 0 |
|  | L2 | 4.2 | 4.4 | 0.2 | 0 | 0 | 0.00000 | 0 | 0 |
|  | L3 | 4.4 | 4.6 | 0.2 | 0 | 0 | 0.00000 | 0 | 0 |
|  | L4 | 4.6 | 4.8 | 0.2 | 0 | 0 | 0.00000 | 0 | 0 |
|  | L5 | 4.8 | 5 | 0.2 | 0 | 0 | 0.00000 | 0 | 0 |
|  | L6 | 5 | 5.2 | 0.2 | 0 | 0 | 0.00000 | 0 | 0 |
|  | L7 | 5.2 | 5.4 | 0.2 | 0 | 0 | 0.00000 | 0 | 0 |
|  | L8 | 5.4 | 5.6 | 0.2 | 0 | 0 | 0.00000 | 0 | 0 |

| Reach (Control  Volume) | BMA | RRM Start | RRM End | BMA miles | Min | Min Likely | Mean | Max  Likely | Max |
| --- | --- | --- | --- | --- | --- | --- | --- | --- | --- |
| RM 8‐10 | L9 | 5.6 | 5.8 | 0.2 | 0 | 0 | 0.00000 | 0 | 0 |
|  | L10 | 5.8 | 6 | 0.2 | 0 | 0 | 0.00000 | 0 | 0 |
|  | R1 | 4 | 4.2 | 0.2 | 0 | 0 | 0.00000 | 0 | 0 |
|  | R2 | 4.2 | 4.4 | 0.2 | 0 | 0 | 0.00000 | 0 | 0 |
|  | R3 | 4.4 | 4.6 | 0.2 | 0 | 0 | 0.00000 | 0 | 0 |
|  | R4 | 4.6 | 4.8 | 0.2 | 0 | 0 | 0.00000 | 0 | 0 |
|  | R5 | 4.8 | 5 | 0.2 | 0 | 0 | 0.00000 | 0 | 0 |
|  | R6 | 5 | 5.2 | 0.2 | 0 | 0 | 0.00000 | 0 | 0 |
|  | R7 | 5.2 | 5.4 | 0.2 | 0 | 0 | 0.00000 | 0 | 0 |
|  | R8 | 5.4 | 5.6 | 0.2 | 0 | 0 | 0.00000 | 0 | 0 |
|  | R9 | 5.6 | 5.8 | 0.2 | 0 | 0 | 0.00000 | 0 | 0 |
|  | R10 | 5.8 | 6 | 0.2 | 0 | 0 | 0.00000 | 0 | 0 |
|  | Total |  |  | 4 | 0 | 0 | 0 | 0 | 0 |
